# Inhaled Nitric Oxide in preterm infants: a systematic review and individual patient data meta-analysis

**DOI:** 10.1186/1471-2431-10-15

**Published:** 2010-03-23

**Authors:** Lisa M Askie, Roberta A Ballard, Gary Cutter, Carlo Dani, Diana Elbourne, David Field, Jean-Michel Hascoet, Anna Maria Hibbs, John P Kinsella, Jean-Christophe Mercier, Wade Rich, Michael D Schreiber, Pimol Srisuparp, Nim V Subhedar, Krisa P Van Meurs, Merryn Voysey, Keith Barrington, Richard A Ehrenkranz, Neil Finer

**Affiliations:** 1 NHMRC Clinical Trials Centre, University of Sydney, Australia; 2 University of California, San Francisco School of Medicine, San Francisco, USA; 3 University of Alabama at Birmingham, School of Public Health, USA; 4 Department of Surgical and Medical Critical Care, Section of Neonatology, Careggi University Hospital of Florence, Florence, Italy; 5 London School of Hygiene and Tropical Medicine, London, UK; 6 Department of Health Science, University of Leicester, Leicester, UK; 7 Neonatology, Maternite Regionale Universitaire, Nancy, France; 8 Case Western Reserve University and Rainbow Babies & Children's Hospital, Cleveland, USA; 9 University of Colorado School of Medicine, Denver, USA; 10 Reanimation Pediatrique Hospital, Paris, France; 11 Division of Neonatology, University of California, San Diego, USA; 12 University of Chicago, Chicago, USA; 13 Division of Neonatology, Mahidol University, Bangkok, Thailand; 14 Neonatal Unit, Liverpool Women's Hospital, UK; 15 Stanford University School of Medicine, USA; 16 Division of Neonatology, Centre Hospitalier Universitaire Ste-Justine, Montreal, Canada; 17 Department of Pediatrics, Yale University School of Medicine, USA

## Abstract

**Background:**

Preterm infants requiring assisted ventilation are at significant risk of both pulmonary and cerebral injury. Inhaled Nitric Oxide, an effective therapy for pulmonary hypertension and hypoxic respiratory failure in the full term infant, has also been studied in preterm infants. The most recent Cochrane review of preterm infants includes 11 studies and 3,370 participants. The results show a statistically significant reduction in the combined outcome of death or chronic lung disease (CLD) in two studies with routine use of iNO in intubated preterm infants. However, uncertainty remains as a larger study (Kinsella 2006) showed no significant benefit for iNO for this combined outcome. Also, trials that included very ill infants do not demonstrate significant benefit. One trial of iNO treatment at a later postnatal age reported a decrease in the incidence of CLD. The aim of this individual patient meta-analysis is to confirm or refute these potentially conflicting results and to determine the extent to which patient or treatment characteristics may explain the results and/or may predict benefit from inhaled Nitric Oxide in preterm infants.

**Methods/Design:**

The Meta-Analysis of Preterm Patients on inhaled Nitric Oxide **(MAPPiNO)** Collaboration will perform an individual patient data meta-analysis to answer these important clinical questions. Studies will be included if preterm infants receiving assisted ventilation are randomized to receive inhaled Nitric Oxide or to a control group. The individual patient data provided by the Collaborators will be analyzed on an intention-to-treat basis where possible. Binary outcomes will be analyzed using log-binomial regression models and continuous outcomes will be analyzed using linear fixed effects models. Adjustments for trial differences will be made by including the trial variable in the model specification.

**Discussion:**

Thirteen (13) trials, with a total of 3567 infants are eligible for inclusion in the MAPPiNO systematic review. To date 11 trials (n = 3298, 92% of available patients) have agreed to participate. Funding was successfully granted from Ikaria Inc as an unrestricted grant. A collaborative group was formed in 2006 with data collection commencing in 2007. It is anticipated that data analysis will commence in late 2009 with results being publicly available in 2010.

## Background

Approximately 8-13% of infants are born prematurely across developed countries. Preterm delivery accounts for 75-80% of all neonatal morbidity and mortality [1,2]. Although survival rates have markedly improved in recent decades, premature infants requiring assisted ventilation are still at significant risk of both pulmonary and cerebral injury.

An estimated 75% of the infants with a birth weight less than 1000 grams develop respiratory distress syndrome (RDS), and nearly 30% are still oxygen dependent at a postmenstrual age of 36 weeks [3]. The commonest definition of chronic lung disease (CLD) is oxygen dependency or respiratory support at 36 weeks postmenstrual age. Infants with severe CLD remain at high risk for pulmonary morbidity and mortality during the first two years of life [4]. In addition, long-term neurodevelopmental impairments associated with cerebral palsy, mental retardation, sensorineural hearing loss, and visual impairment are frequently observed in infants with CLD [5,6]. The incidence rate of these neurodevelopmental impairments increases with decreasing birth weight. Neonates with birth weights of 1501 to 2500 grams have an 8% incidence, compared with a 25% rate in infants born weighing less than 1000 g [7].

Nitric Oxide (NO) relaxes vascular smooth muscle by activating guanyl cyclase and leading to the production of cyclic GMP [8]. The first experimental study in immature lambs reported that exogenous inhaled NO (iNO) selectively increased pulmonary blood flow and reduced pulmonary artery pressure [9]. Studies in adults also show that iNO improves ventilation/perfusion mismatch by selective pulmonary vasodilation [10,11]. A meta-analysis showed iNO improved oxygenation in approximately 50% of full term or near term infants with pulmonary hypertension and hypoxic respiratory failure. There was a significant reduction in the incidence of death or requirement for extracorporeal membrane oxygenation (ECMO). However, the authors emphasized that results of iNO in term infants cannot be extrapolated to the premature infants because of different pathophysiology, different inclusion criteria, and different outcomes assessed [8]. Although initially investigated for its pulmonary vasodilating effect, it has become clear that the potential pulmonary effects of iNO are multiple and complex. There are pro-oxidant and anti-oxidant effects [12] and in experimental animal models of neonatal chronic lung disease, pulmonary structure and function are protected by iNO [13], suggesting that there are direct effects which could potentially reduce chronic lung disease.

Several randomized controlled trials have been conducted in preterm infants to determine whether iNO reduces the rates of death and/or chronic lung disease [14-26]. The results of these studies appear contradictory. Some studies have shown a reduction in lung injury, one has shown a reduction in cerebral injury, and several others have shown no effect. The different patient characteristics and different trial characteristics within these trials may explain this difference.

### Summary of aggregate data systematic review in 2007

The most recent Cochrane review includes 11 studies and 3,370 participants [27]. These studies differ not only in their design and intervention, but in the eligible patient populations. For example, Schreiber 2003 and Kinsella 2006 studied the routine use of inhaled NO in all intubated preterm infants who had a relatively low oxygen requirement and severity of illness at intubation [22,20]. The studies by Van Meurs et al [26] and the INNOVO study group from the UK [18], in contrast, only enrolled patients with severe hypoxic respiratory failure and as a result had extremely high incidences of the combined outcome of death or CLD. The entry criteria also differed with most studies enrolling infants in the first 48 hours, but Ballard et al [14,15] enrolled infants between 7 and 21 days who were at high risk of developing CLD. Hence, the review authors divided these trials into three categories based on different entry criteria: entry in the first three days of life according to oxygenation criteria, routine use in intubated preterm babies and later enrolment based on an increased risk of CLD. The results showed there was a marginally significant reduction in death or CLD at 36 weeks with a relative risk of 0.91 (95% CI 0.84-0.99) in studies with routine use of iNO in intubated preterm infants. However, trials of early treatment of infants based on oxygenation criteria or of later enrolment based on the risk of CLD did not demonstrate significant benefit of iNO for the primary end point of death or CLD at 36 weeks, when analyzed according to standard aggregate data meta-analytic techniques. Almost all information from late enrolled babies is derived from a single large study (Ballard et al) which reported a significant reduction in the outcome of death or CLD [14,15]. These data were analyzed using a new method (multiple outputation) to account for potential confounding effects of enrolling infants from multiple gestations, of whom only the first eligible infant was randomized. One way in which to confirm or refute these results and to determine whether certain patient or treatment characteristics may predict benefit from inhaled Nitric Oxide in premature infants is by means of an individual patient data meta-analysis.

The advantages of an individual patient data meta-analysis over a meta-analysis based on aggregate data are as follows:

• It is possible to ensure uniformity in defining patient characteristics and outcome measures.

• Such analysis can assess the relationship between patient-level characteristics and treatment effect, resulting in a differentiation of the treatment effect according to risk profiles.

• A more accurate assessment of how trial characteristics may affect response is feasible.

• Information on long-term outcome can be updated.

• It is possible to develop predictive models using multivariate regression analyses.

### Objectives

1. To determine whether inhaled Nitric Oxide in preterm infants receiving assisted ventilation improves survival without morbidity, specifically without CLD or major neurological injury.

2. To determine whether the effects of inhaled Nitric Oxide differ according to the risk profile of the patient in terms of gestational age at birth, severity of illness, antenatal steroid use, postnatal age at the time of randomization, ventilation mode at randomization, administration of exogenous surfactant, inhaled Nitric Oxide dosage and duration of administration.

## Methods

### Inclusion criteria for studies

Study design: Studies will be included if they are randomized controlled trials.

Participants: Preterm infants (less than 37 weeks gestation) receiving assisted ventilation. Intervention: Inhaled Nitric Oxide compared to control.

### Search strategy

The standard search strategy of the Cochrane Neonatal Review Group will be used to identify potentially eligible studies. This involves extensive searching of bibliographic databases such as MEDLINE, EMBASE.com, *The Cochrane Controlled Trials Register *and Healthstar from 1985 to 2009. The terms "Nitric Oxide" and "newborn" will be used and the search limited to clinical trial. The abstracts of the Pediatric Academic Societies will also be searched from 2000 to 2009. In addition, all members of the Collaborative Group will be asked to notify the group of any unpublished trials of which they are aware. See Table 1 for the citations of known eligible studies at October 2009 and Table 2 for a description of these studies.

**Table 1:** MAPPiNO Collaboration: citations for eligible trials as at October 2009

Trial identifier	Main citation
Ballard 2006	Ballard RA, Truog WE, Cnaan A, et al. Inhaled Nitric Oxide in preterm infants undergoing mechanical ventilation. *New England Journal of Medicine *2006; 355(4):343-53.

Dani 2006	Dani C, Bertini G, Pezzati M, et al. Inhaled Nitric Oxide in very preterm infants with severe respiratory distress syndrome. *Acta Pediatrica *2006; 95: 1116-1123.

Hascoet 2005	Hascoet JM, Fresson J, Claris O, et al. The safety and efficacy of Nitric Oxide therapy in premature infants. *Journal of Pediatrics *2005; 146 (3):318-23.

INNOVO 2005	INNOVO. Neonatal ventilation with inhaled Nitric Oxide versus ventilatory support without inhaled Nitric Oxide for preterm infants with severe respiratory failure: the INNOVO multicentre randomized controlled trial. *Pediatrics *2005; 115 (4):926-36.

Kinsella 1999	Kinsella JP. Inhaled Nitric Oxide in premature neonates with severe hypoxaemic respiratory failure: a randomised controlled trial. *Lancet *1999; 354(9184):1061.

Kinsella 2006	Kinsella JP, Cutter GR, Walsh WF, et al. Early inhaled Nitric Oxide therapy in premature newborns with respiratory failure. *New England Journal of Medicine *2006; 355(4):354-64.

Mercier 1999	Mercier JC, Thebaud B, Onody P. Early compared with delayed inhaled Nitric Oxide in moderately hypoxaemic neonates with respiratory failure: a randomised controlled trial. *Lancet *1999; 354(9184):1066.

Schreiber 2003	Schreiber MD, Gin-Mestan K, Marks JD, et al. Inhaled Nitric Oxide in Premature Infants with the Respiratory Distress Syndrome. *New England Journal of Medicine *2003; 349(22):2099-107.
	Mestan KKL, Marks JD, Hecox K, et al. Neurodevelopmental Outcomes of Premature Infants Treated with Inhaled Nitric Oxide. *New England Journal of Medicine *2005; 353(1):23-32.

Srisuparp 2002	Srisuparp P, Heitschmidt M, Schreiber MD. Inhaled Nitric Oxide therapy in premature infants with mild to moderate respiratory distress syndrome. *J Med Assoc Thai *2002; 85(Suppl 2): S469-S478.

Su 2008	Su PH, Chen JY. Inhaled Nitric Oxide in the management of preterm infants with severe respiratory failure. *Journal of Perinatology *2008; 28: 112-116.

Subhedar 1997	Subhedar NV, Ryan SW, Shaw NJ. Open randomised controlled trial of inhaled Nitric Oxide and early dexamethasone in high risk preterm infants. *Arch Dis Child Fetal Neonatal Ed *1997; 77(3): F185-F190.
	Subhedar NV, Shaw NJ. Changes in oxygenation and pulmonary haemodynamics in preterm infants treated with inhaled Nitric Oxide. *Arch Dis Child Fetal Neonatal Ed *1997; 77(3):F191-F197.

Van Meurs 2005	Van Meurs KP, Wright LL, Ehrenkranz RA, et al. Inhaled Nitric Oxide for Premature Infants with Severe Respiratory Failure. *New England Journal of Medicine *2005; 353(1):13-22.

EUNO 2008 (completed RCT awaiting publication)	JC Mercier, H. Hummler, X Durrmeyer, et al. The effects of inhaled Nitric Oxide on the development of bronchopulmonary dysplasia in preterm infants: the 'EUNO' multicentre randomised clinical trial. Abstract: *European Academy of Pediatrics*, Nice, France, October 25, 2008.

**Table 2:** MAPPiNO Collaboration: description of eligible trials as at October 2009

Study	Participants	Intervention	Primary outcome
Ballard 2006	582 infants <1250 g and <32 wks on assisted ventilation at 7-21 days (or, if <800 g, on CPAP)	Inhaled NO at 20 ppm initial dose for 48 to 96 hours, then dose subsequently decreased to 10, 5, and 2 ppm at weekly intervals, with a minimum treatment duration of 24 days	Survival without BPD at 36 wks postmenstrual age

Dani 2006	40 infants <30 wks ventilated with severe RDS: FiO_2 _>0.5 and arterial-alveolar oxygen ratio < 0.15 despite surfactant treatment	Inhaled NO at 10 ppm for 4 hours followed by 6 ppm. Weaning (decrease by 2 ppm every 3 hrs) started at 72 hrs or when the infant was extubated or when the FiO_2 _<0.3 with a mean airway pressure <8 cmH_2_O	Death or BPD (oxygen requirement at 36 weeks postconceptional age) in survivors

Hascoet 2005	860 infants <32 wk enrolled at birth; n = 145 infants were eligible for study gas as had hypoxic respiratory failure (defined as need for mechanical ventilation, FiO_2_>0.40 and arterio-alveolar O_2 _ratio <0.22) at 6-48 hrs age	Inhaled NO was administered starting at 5 ppm, with adjustments allowed depending on response up to a maximum of 10 ppm. Subjects were allowed to receive (unblinded) iNO in either group if they developed refractory hypoxemia.	Intact survival at 28 days (defined as alive without need for oxygen supplementation or IVH >grade 1 or refractory hypoxaemia (need for 100% oxygen with PaO_2_<50 mmHg) and PCO_2 _<50 mmHg)

INNOVO 2005	108 preterm infants (<34 wks) less than 28 days of age with severe respiratory failure requiring ventilator support and have had surfactant when appropriate	Inhaled NO usually at 5 ppm, up to 40 ppm based on response criteria (satisfactory response: increase in PaO_2 _>22.5 mmHg after 15 minutes iNO)	1) Death or severe disability at 1 year corrected age; and 2) Death before discharge or continued oxygen need at 36 wks pma and/or at expected date of delivery

Kinsella 1999	80 preterm infants (</= 34 weeks) aged 7 days or less, with a/A ratio <0.1 on two sequential arterial blood measurements despite mechanical ventilation and surfactant treatment	Inhaled NO at 5 ppm for 7 days after which periods of no study gas were tried; threshold criteria for gas re-start was an increase of >/=15% in OI; maximum treatment duration was 14 days	Survival to discharge

Kinsella 2006	793 preterm infants < 34 wks, with respiratory failure needing assisted ventilation in first 48 hours of life	Inhaled NO at 5 ppm for 21 days or until extubation	Death or BPD (need for supplemental oxygen or mechanical ventilation at 36 wks pma and abnormal CXR)

Schreiber 2003	207 infants < 34 wks and < 2000 g birth weight, < 72 hours of age, and intubated/ventilated for RDS, having had exogenous surfactant	Inhaled NO starting at 10 ppm for 12-24 hrs, then 5 ppm for 6 days, then weaned by 1 ppm every 6 hrs if PaO_2 _did not decrease by more than 15% until extubation; 2 × 2 factorial trial of iNO vs control gas and HFOV vs CMV	Death or CLD (supplemental oxygen and CXR showing persistent parenchymal lung disease at 36 weeks pma) among surviving infants

Srisuparp 2002	34 infants < 2000 g, ventilated after surfactant with an arterial catheter and less than 72 hours of age + satisfying severity of illness criterion: OI >4 if birthweight<1000 g; >6 if 1001-1250 g; >8 if 1251-1500 g; >10 if 1501-1750 g; and >12 if 1751-2000 g birthweight	Inhaled NO at 20 ppm for 6-12 hrs, then reduced to 10 ppm, and weaned to 5 ppm in the next 12 hrs; weaning tolerated if PaO_2_did not decrease by more than 15%; once 5 ppm achieved, weaning was attempted at 1 ppm a time as tolerated until gas discontinued; maximum duration allowed was 7 days	Severe intraventricular hemorrhage (grade 3 or 4)

Subhedar 1997	42 preterm infants, < 32 wks, assessed at 96 hrs age for: mechanical ventilation since birth, had received surfactant, and high risk of developing CLD using a modified prediction score	Inhaled Nitric Oxide at 20 ppm for 2 hrs then weaned according to response criteria (positive response: decrease in OI by >=25% or reduction in FiO_2 _of >=0.10) by 5 ppm increments every 15 mins until 5 ppm level for further 72 hrs, then weaned off; 2 × 2 factorial trial of iNO vs control and IV dexamethasone vs control	Death before discharge or CLD (oxygen dependency for at least 28 days and beyond 36 wks pma with abnormal CXR)

Van Meurs 2005	420 preterm infants, < 34 weeks, 401-1500 g birthweight, assisted ventilation, OI >=10 on two consecutive blood gases 30 min -12 hrs apart at least 4 hrs after surfactant	Inhaled Nitric Oxide initially at 5-10 ppm; weaning commenced 10-14 hrs after initiation according to response criteria (change in PaO_2_); at 30 min intervals; maximum duration was 336 hours	Death or BPD at 36 wks in survivors

EUNO 2008	800 preterm infants <29 wks, birthweight >=500 g requiring either surfactant or CPAP >4 cmH_2_O with FiO_2 _>0.3 to maintain SpO_2 _≥ 85%	Inhaled NO 5 ppm for minimum 7 to maximum of 21 days if still requiring respiratory support (including CPAP use)	Survival without BPD at 36 wks post conceptional age

a/A: arterial/alveolar oxygen ratio

BPD: bronchopulmonary dysplasia

CLD: chronic lung disease

CMV: continuous mechanical ventilation

CPAP: continuous positive airway pressure

CXR: chest X ray

FiO_2_: fraction of inspired oxygen

g: grams

HFOV: high-frequency oscillatory ventilation

hrs: hours

iNO: inhaled Nitric Oxide

OI: oxygenation index

pma: postmenstrual age

ppm: parts per million

RDS: respiratory distress syndrome

SpO_2_: oxygen saturation

wks: weeks

### Data management

De-identified individual patient data provided by the Collaborators (see Additional file 1 for the suggested coding sheet and Additional file 2 for the data provision form) will be recoded as required and stored in an electronic database at the Data Coordination Centre. Electronic data will be located on a secure password-protected network server. Copies of hardcopy data will be stored in locked filing cabinets until converted into electronic format, and will then be securely destroyed. Only authorized personnel will have access to this data.

The data will be checked with respect to range, internal consistency, consistency with published reports and missing items. Trial details such as randomization, methods and intervention details will be crosschecked against published reports, trial protocols and data collection sheets. Inconsistencies or missing data will be discussed with the individual trialists, and attempts will be made to resolve any discrepancies by consensus. Each trial will be analyzed individually and the resulting analyses and trial data will be sent to the trialists' for verification.

### Data items to be requested from the trialists

Trial-level information: obtained from the trial protocol and/or the trialists

1. Dates the trial opened and closed to accrual

2. Number of patients randomized

3. Informed consent procedures

4. Methods of random allocation

5. Stratification factors used

6. Methods of allocation of concealment

7. Blinding of outcome assessment

8. Details of the intervention in the experimental arm

• Inhaled Nitric Oxide concentration

• Durations allowed

• Protocol for weaning

• Target oxygen saturation range

• Target blood gas range

9. Details of the intervention in the control arm

• Target oxygen saturation range

• Target blood gas value range

10. Criteria for permitted crossover from the assigned treatment

11. Criteria for failure of assigned treatment

12. Details of surfactant replacement therapy, if determined by the protocol

13. Criteria for postnatal treatment with systemic corticosteroids

Patient-level information: characteristics at study entry

1. Unique identification coded for anonymity

2. Time or postnatal age at intubation

3. Time or postnatal age at randomization

4. Gestational age at birth

5. Birth weight

6. Antenatal corticosteroid therapy, complete (>24 hours) or not

7. Sex

8. Race

9. Inborn/outborn status

10. Type of respiratory support (endotracheal tube - conventional or high frequency, nasal CPAP, other)

11. Ventilator rate at time of randomization

12. Components of a respiratory severity score at time of randomization (PIP, MAP and FiO_2_)

13. PaCO_2 _at time of randomization

14. PaO_2 _or oxygen saturation at time of randomization

15. Use of surfactant replacement therapy

16. Type of surfactant used (natural or synthetic)

17. Postnatal age at first dose of surfactant

18. Prophylactic indomethacin

19. PDA

20. Postnatal treatment with systemic corticosteroids

21. Worst pre-randomization cranial ultrasound result

Patient-level information: data on actual study intervention

1. Study gas assigned

2. Highest and starting dosage received

3. Duration of therapy

4. Any re-treatment after study period

5. Other drugs in same pathway

6. Change in ventilation mode during therapy (CMV to HFV or vice versa)

7. Failure of assigned treatment

8. Need for treatment crossover during study period

Patient-level information: data on neonatal outcome

1. Mortality and age at death

2. Duration of mechanical ventilation

3. Duration of oxygen therapy

4. Duration of any respiratory support (mechanical ventilation, CPAP or oxygen)

5. Gross pulmonary air leak (pneumothorax or other gross air leak including pneumomediastinum, pneumopericardium or pneumoperitoneum)

6. Pulmonary interstitial emphysema

7. Pulmonary hemorrhage

8. Worst post-randomization cranial ultrasound result (including acute periventricular hemorrhage such as subependymal, intraventricular or intracerebral)

9. Ventricular dilatation at any stage

10. Cystic periventricular leukomalacia

11. Worst stage of retinopathy of prematurity (ROP)

12. Threshold ROP

13. Surgical or laser therapy for ROP

14. Duration of hospital stay

15. Home oxygen therapy

### Planned analyses

Binary outcomes will be analyzed using log-binomial regression models adjusting for trial differences by including the trial variable in the model specification. Exponentiating the parameter estimate for treatment from a log-binomial regression model produces a relative risk for treatment.

Outcomes between siblings from multiple births are highly correlated and must be accounted for in the analysis. There are two main methods of adjusting for such 'clustered' data which will be utilized in this study. The primary method will be the multiple outputation approach. This method involves randomly selecting one patient from each sibling cluster and running the analysis on this set of independent data to obtain an estimate of the effect  and an estimate of its variance . This process is then repeated N times. The average of the estimated  's from each iteration  is used as the estimate of the overall effect and an estimate of the variance is given by the average of the variances  minus the variance of the effect estimates [28]. This method involves no additional assumption about correlations between siblings and therefore avoids problems of numerical instability when fitting the models.

As a sensitivity analysis, additional methods of accounting for correlations within the data will be used on the primary outcomes. For example generalized estimating equations (GEE) may be used to analyse the two main endpoints of interest (death or CLD and neurological injury). The GEE model is a repeated measures model for binary outcomes which accounts for the correlation between siblings.

Continuous normally distributed endpoints will be analyzed using a linear fixed effects model. Additionally the treatment by trial interaction will be assessed to investigate possible heterogeneity of treatment effects [29]. The overall estimated mean and standard deviation within each treatment group will be presented along with the mean difference in treatment effect and its 95% confidence interval with p value. If the data do not meet the assumptions for the model then transformations or alternative models will be investigated.

The MAPPiNO Collaboration aims to collect all the available worldwide individual patient data for preterm infants randomized in clinical trials assessing the effect of inhaled Nitric Oxide. Currently there is a commitment to provide data from 3298 infants in 11 trials (92% of worldwide data). A sample size of this magnitude would have at least 89% power to detect relative changes of 10% in the risk of death or chronic lung disease (main outcome) for the treated group across a range of plausible baseline event rates (55-75%) with a two sided α = 5% and 1:1 ratio for number treated to control.

A summary of the planned analyses is listed below. A detailed analysis plan is outlined in a separate document and available upon request.

#### Outcomes to be analyzed

The main analyses comparing the effect of inhaled Nitric Oxide to standard therapy will be undertaken for the outcomes listed below. The planned subgroups and sensitivity analyses will be restricted to the main outcomes.

a. Primary outcomes

• Death or chronic lung disease (CLD) using the best available definition (alive and oxygen dependent at 36 weeks postmenstrual age (PMA) if calculable, otherwise trialists' own definition)

• Severe adverse neurological event after randomization (intracranial hemorrhage (IVH) grade III or IV, or cystic periventricular leukomalacia (PVL) or other pathologies such as periventricular echodensity, periventricular cysts, ventriculomegaly or hydrocephalus)

b. Secondary outcomes

• Death at any time, by 36 weeks PMA and at discharge

• Severe IVH (grade III or IV) with and without adjustment for baseline IVH severity

• Survivors without CLD at 36 weeks PMA

• Severe adverse neurological event (IVH grade III or IV, or PVL or other pathologies such as periventricular echodensity, periventricular cysts, ventriculomegaly or hydrocephalus) with and without adjustment for baseline status

• Postnatal steroid use

• Gross pulmonary air leak (at least one of the following: pneumothorax, pneumomediastinum, pneumoperitoneum or pneumopericardium)

• Pulmonary hemorrhage

• Failure of assigned treatment

• Duration of oxygen therapy

• Duration of hospital stay

• Home oxygen therapy

• Severe retinopathy of prematurity (ROP stage >=3; surgical or laser therapy for ROP)

• Postmenstrual age when ETT ceased

• Postmenstrual age when respiratory support ceased

• Postmenstrual age when discharge from hospital

c. Additional outcome

• In addition, CLD at 36 and 28 weeks postnatal age, CLD as classified by the trialist and CLD classified using the best available definition (alive, oxygen dependent at 36 weeks or trialists' own definition) will be assessed. However it is noted that since these outcomes can only be assessed for babies who survive to these time points, these do not constitute ITT analyses nor are they randomized comparisons.

#### Planned subgroup analyses

One of the strengths of individual patient data meta-analyses is that they allow subgroup analyses to be performed. For both main endpoints, subgroup analyses will be undertaken to determine if the effect of iNO treatment differs depending on patient-level characteristics. That is, are there any particular patient characteristics that determine who may benefit from inhaled Nitric Oxide. These analyses will allow us to take into account each individual infant's own characteristics rather than relying on summary measures of the average risk profile of all patients in an individual trial. We will examine the following characteristics:

1. Gestational age at birth

2. Birth weight

3. Postnatal age at entry into the study

4. Severity of lung disease (at study entry)

5. Inhaled Nitric Oxide dosage

6. Duration of therapy

7. Measure of iNO exposure (incorporating dose and duration)

8. Antenatal steroid administration

9. Postnatal steroids administration before initiation of iNO

10. Ventilation mode at randomization

11. Administration of exogenous surfactant

12. Presence of pulmonary hypertension

13. Multiple birth

14. Race

#### Planned sensitivity analyses

The following sensitivity analyses will be performed for the main outcomes to compare the overall estimates of treatment effect calculated on all data, to estimates based on subsets of the data with the following data removed:

• Trials with <50 study patients

• Trials with inadequate concealment of allocation or blinding

• Trials with high rates of patient exclusions (>40%)

#### Planned additional analyses

Multivariable regression models will be developed to determine which patient-level characteristics are predictive of the main outcomes.

### Ethical considerations

Participants in the individual trials have previously given informed consent to participate in their respective trial. The data for this project are to be used for the purpose for which they were originally collected and are available through an agreement between all trialists of the collaborative group. These trialists remain the custodian of their original individual trial data at all times.

### Project management

Membership of the MAPPiNO Collaboration will include representative(s) from each of the trials contributing data to the review with an accompanying project coordination and data management structure as described in this section.

The membership and responsibilities of each of these management groups are as follows:

### Steering Group

The Steering Group will be responsible for project management decisions and will meet approximately 4-6 times per year, usually via teleconference. Membership: N Finer^1 ^(chair), K Barrington^2^, R Ehrenkranz^3^, W Rich^1^, L Askie^4 ^(data coordination manager), A Carberry^4^(data manager).

^1 ^Division of Neonatology, University of California, San Diego, USA;

^2 ^Division of Neonatology, Centre Hospitalier Universitaire Ste-Justine, Montreal;

^3 ^Department of Paediatrics, Yale University School of Medicine, USA;

^4 ^NHMRC Clinical Trials Centre, University of Sydney, Australia.

### Advisory Group

The aim of the Advisory Group is to facilitate representative input from the Collaborative Group to the Steering Group if this is warranted. Membership of the Advisory Group will be at the invitation of the Steering Group.

### Collaborative Group

All potentially eligible trialists will be contacted and invited to become members of the Collaborative Group. The corresponding author for each study will be contacted in the first instance. If there is no response, the associated statistician, data manager and/or other authors will be contacted. This process will be updated annually for the duration of the project, to ensure that new trialists are offered the opportunity to join the project and contribute their data.

### Data Coordination Centre

The project will be coordinated from the NHMRC Clinical Trial Centre, University of Sydney, NSW, Australia. The Data Coordination Centre will be responsible for the daily management of the project including correspondence, newsletter production, maintaining current trialist contact information, meeting and teleconference organisation, and receipt, storage and analysis of project data as directed by the Collaborative Group via the Steering Group.

### Collaborators' meetings

All members of the Collaboration, including the Steering Group, the Advisory Group, and representatives of each participating trial, will be invited to attend regular collaborators' meetings. The meetings will be designed to allow maximum input from the participating trialists into the design, conduct, analysis and reporting of the project's results.

### Publication policy

The results of the project's analyses will be presented to, and discussed with, the Collaborative Group before presentation and publication. The main manuscript will be prepared by the Steering Group, and circulated to the Collaborative Group for comment and revision. The revised draft paper then will be circulated to all members of the Collaborative Group for comment and agreement before publication. Publications using these data will be authored on behalf of the Meta Analysis of Preterm Patients on inhaled Nitric Oxide (MAPPiNO) Collaboration, either with specific named authors, or on behalf of the Collaboration as a whole. Names of other participating Collaborators will be acknowledged in an appropriate section of the manuscript.

## Discussion

A recently updated meta-analysis [27] showed that inhaled Nitric Oxide marginally reduced the incidence of death or CLD as well as severe brain damage in two studies where iNO was used routinely for mildly sick preterm infants. However, uncertainty remains as a larger study (Kinsella 2006) showed no significant benefit for iNO. Trials including very ill infants or with later iNO treatment using standard meta-analytic techniques did not demonstrate a significant effect of iNO. Using techniques to correct for the possible confounding effects of having infants from multiple gestations enrolled in the same group (multiple outputation and general estimating equations) one trial (Ballard 2006) did show a significant reduction in the combined outcome of death or CLD with treatment which commenced between 7 and 21 days. The best way to answer these remaining questions is to utilize existing individual patient data from all infants enrolled in these trials. This approach has been described as the 'gold standard' of systematic review methodology as it allows for more powerful and flexible analysis of both subgroups and outcomes. The MAPPiNO Collaboration has been formed to undertake a systematic review of all available trials, with meta-analysis based on individual patient data, to answer these important clinical questions. Provision of data by the participating Collaborators commenced in 2007 and results will be ready for presentation in 2010.

## Competing interests

The authors declare that they have no competing interests.

## Authors' contributions

LMA participated in the design and coordination of the study and drafted the manuscript. RAB, GC, CD, DE, DF, J-MH, AMH, JPK, J-CM, WR, MDS, PS, NVS, KPVM participated in the design of the study and helped to draft the manuscript. MV participated in the design of the study, performed the statistical analysis and helped to draft the manuscript. KB, RAE and NF conceived of the study, participated in its design and coordination and helped to draft the manuscript. All authors read and approved the final manuscript.

## Authors' information

The named authors worked on this paper on behalf of the Meta-Analysis of Preterm Patients on inhaled Nitric Oxide (MAPPiNO) Collaboration.

## Pre-publication history

The pre-publication history for this paper can be accessed here:

http://www.biomedcentral.com/1471-2431/10/15/prepub

## Supplementary Material

Additional file 1 **Suggested coding sheet**. table listing variables collected and suggested coding. Click here for file

Additional file 2 **Data provision form**. collection form for trial level data, data provision procedure. Click here for file
