# Supplementary material for: Inhaled Nitric Oxide in preterm infants: a systematic review and individual patient data meta-analysis
Source: BMC Pediatr. 2010 Mar 23;10:15. doi: 10.1186/1471-2431-10-15 (PMC2860486; doi:10.1186/1471-2431-10-15)
Supplement: Additional file 1 — Suggested coding sheet. table listing variables collected and suggested coding. [file 1471-2431-10-15-S1.PDF]

MAPPiNO

Meta Analysis of Preterm Patients on inhaled Nitric Oxide Collaboration

If possible, please use the suggested coding below when submitting your trial data

Enrolment characteristics

Variable name	Variable coding
Unique patient identifier	Unique patient ID used within your trial (anonymous)
Date of intubation*	Date of intubation
Time of intubation[#]	Time of intubation
Date of randomization*	Date of randomization
Time of randomization[#]	Time of randomization
Date of birth*	Date of birth
Time of birth[#]	Time of birth
Gender	1=male; 2=female; 9=unknown
Gestational age at birth	In completed weeks using best estimate; 99=unknown
Birth weight	Birth weight, in grams; 9999=unknown
multiple birth	0=singleton; 1=multiple; 9=unknown
Inborn / outborn status^	1=inborn; 2=outborn; 9=unknown
Race	1=white, not Hispanic; 2=Hispanic; 3=black, not Hispanic; 4=Asian or Pacific Islander;5=American Indian or Alaska Native; 6=Other; 9= Unknown
Type of respiratory support	1= conventional ETT; 2= high frequency ETT♥; 3=nasal CPAP; 4=other°; 9=Unknown
Antenatal corticosteroids	0=no; 1=yes; 9=unknown
PIP at time of randomisation	In cm H2O
MAP at time of randomisation	In cm H2O
PaCO2 at time of randomisation	In mmHg
FiO2 at time of randomisation	In percent (eg. 67%)
PaO2 at time of randomisation	In mmHg
SpO2 at time of randomisation	In percent (eg. 41%)
Surfactant replacement therapy	0=No; 1=Yes; 9=Unknown
Type of Surfactant	1=Natural; 2=Synthetic; 3=both; 4=others; 9=Unknown
Date at first dose of surfactant*	Date at first dose of surfactant
Time at first dose of surfactant[#]	Time at first dose of surfactant
Prophylactic indomethacin	0=No; 1=Yes; 9=Unknown
Patent ductus arteriosus	0=No; 1=Yes; 9=Unknown
Postnatal steroids	0=No; 1=Yes; 9=Unknown
Primary respiratory diagnosis	In text
Pulmonary hypertension	0=No; 1=Yes; 9=Unknown
Pulmonary vasodilator therapy	0=No; 1=Yes; 9=Unknown
Date* of worst head ultrasound	Date of worst head ultrasound
Worst intracranial haemorrhage pre-randomisation	0=None; 1= Grade I; 2= Grade II; 3=Grade III; 4=Grade IV; 9=Unknown
Cystic periventricular leukomalacia	0=No; 1=Yes; 9=Unknown
Periventricular echodensity	0=No; 1=Yes; 9=Unknown

Enrolment characteristics

Variable name	Variable Coding
Periventricular cysts	0=No; 1=Yes; 9=Unknown
Ventriculomegaly	0=No; 1=Yes; 9=Unknown

Study intervention details

Study gas assigned	1=inhaled NO (iNO); 2=control
Starting dose iNO received♓	In ppm (for control group: code as 9999)
Highest dose iNO received♓	In ppm (for control group: code as 9999)
Date study gas started*♓	Date of study gas started
Time study gas started[#]♓	Time of study gas started
Date study gas ceased*♓	Date of study gas ceased
Time study gas ceased[#]♓	Time of study gas ceased
Total days of study gas ♓	In days (for: control group code as 9999)
Other drugs in same pathway (sildenafil etc)	0=No; 1=Yes; 9=Unknown
If yes, please specify	In text
Type of respiratory support used after study initiation and before study discontinuation	1= conventional ETT; 2= high frequency ETT♥; 3=nasal CPAP; 4=other°; 9=Unknown
Change in ventilation mode during study period §	0=No; 1=Yes; 9=Unknown
Failure of assigned treatment	0=No; 1=Yes; 9=Unknown
If yes, give reason for failure	In text
Date of treatment failure*	Date of treatment failure
Time of treatment failure [#]	Time of treatment failure
Need for treatment crossover during study period	0=No; 1=Yes; 9=Unknown
Received 'open label' iNO after study period	0=No; 1=Yes; 9=Unknown

Infants outcomes (after randomisation)

Date of death*	Date of death (if alive/unknown, leave in blank)
Cause(s) of death	In text
Date of extubation*	Date of extubation
Time of extubation[#]	Time of extubation
On ventilatory support at 28 days	0=No; 1=Yes; 9=Unknown
On ventilatory support at discharge home	0=No; 1=Yes; 9=Unknown
Days of mechanical ventilation	Days; 9999=unknown
Days of continuous positive airway pressure (CPAP)	Days; 9999=unknown

Please turn over for further variables and notes:

For further information, contact: mappino@ctc.usyd.edu.au Phone: +61 (0)2 9562 5028

MAPPiNO

Meta Analysis of Preterm Patients on inhaled Nitric Oxide Collaboration

If possible, please use the suggested coding below when submitting your trial data

Infants outcomes (after randomisation)		***Infants outcomes (after randomisation)***	
**Variable name**	**Variable coding**	**Variable name**	**Variable Coding**
Supplemental oxygen at discharge home	0=No; 1=Yes; 9=Unknown	Threshold ROP	0=No; 1=Yes; 9=Unknown
Days of supplemental oxygen	Days; 9999=unknown	Surgical/laser therapy for ROP	0=No; 1=Yes; 9=Unknown
Pneumothorax	0=No; 1=Yes; 9=Unknown	Date of discharge to home*	Date of discharge
Other gross pulmonary air leak¶	0=No; 1=Yes; 9=Unknown	Duration of hospital stay	Days; 9999=unknown
Pulmonary haemorrhage	0=No; 1=Yes; 9=Unknown	Home oxygen therapy	0=No; 1=Yes; 9=Unknown
Pulmonary interstitial emphysema	0=No; 1=Yes; 9=Unknown	***Long term outcomes***	
Chronic lung disease¥	0=No; 1=Yes; 9=Unknown		
Postnatal steroids after initiation of study gas	0=No; 1=Yes; 9=Unknown	Cerebral palsy†	0=No; 1=Yes; 9=Unknown
If yes, total days of postnatal steroid treatment	Days; 9999=unknown	GMFCS level†	1=level 1; 2=level 2; 3=level 3; 4=level 4; 5=level 5; 9=Unknown
Date* of worst head ultrasound	Date of worst head ultrasound	Bayley Mental Development Index II (MDI) score♣	Actual scores; 9999=unknown
If date of worst head ultrasound was not available, please provide postnatal days instead	Day of life of worst head ultrasound	Bayley Psychomotor Developmental Index II (PDI)♣	Actual scores; 9999=unknown
Worst intracranial haemorrhage	0=No; 1= Grade I; 2= Grade II; 3=Grade III; 4=Grade IV; 9=Unknown	Deafness♦	0=No; 1=Yes; 9=Unknown
Cystic periventricular leukomalacia	0=No; 1=Yes; 9=Unknown	Blindness♠	0=No; 1=Yes; 9=Unknown
Periventricular echodensity	0=No; 1=Yes; 9=Unknown	Corrected age when long term growth measures collected*	in weeks; 9999=unknown
Periventricular cysts	0=No; 1=Yes; 9=Unknown	Weight	In grams; 9999=unknown
Ventriculomegaly	0=No; 1=Yes; 9=Unknown	Length / Height	In centimetres; 9999=unknown
Hydrocephalus	0=No; 1=Yes; 9=Unknown	Head circumference	In centimetres; 9999=unknown
Worst stage of Retinopathy of Prematurity (ROP)	0=No; 1=stage 1; 2=stage 2; 3=stage 3; 4=stage 4; 9=Unknown		

Notes:

* Dates should be in the format: dd/mm/yyyy or dd/mm/yy; if date unknown, please leave blank

\# Time format should be in 24 hour time: 20:37 (= 8:37pm); if time unknown, please leave blank

^ Inborn: Infant born at a hospital with neonatal intensive care facilities where the child spent most of the first week of life; Outborn: born at a hospital without neonatal intensive care facilities

¥ Chronic lung disease (CLD), as defined in your trial

§ From conventional mechanical ventilation (CMV) to high frequency ventilation (HFV) or vice versa

ϰ Complete for iNO group only: for control group leave dates / times blank, otherwise code as indicated

¶ Pneumomediastinum or pneumopericardium or pneumoperitoneum

† Ideally defined as Gross Motor Function Classification System (GMFCS) level, or as defined in your trial. Please refer to the following citation: Palisano R et al. Gross Motor Function Classification System for Cerebral Palsy. *Dev Med Child Neurol* 1997; 39: 214-23.

♣ Bayley N. Bayley scales of infant development. 3rd edn. San Antonio, TX: The Psychological Corporation, 1993

♦ Requiring hearing aids in either ear, or too deaf to benefit from a hearing aid, or as defined in your trial

♠ Cannot fixate or is legally blind (<3/60) in both eyes, or as defined in your trial

♥ Types of high frequency ventilation: HFOV, HFJV, HFFI and HFPPV

° If others, please specify the ventilation mode used in text
