# Supplementary material for: Inhaled Nitric Oxide in preterm infants: a systematic review and individual patient data meta-analysis
Source: BMC Pediatr. 2010 Mar 23;10:15. doi: 10.1186/1471-2431-10-15 (PMC2860486; doi:10.1186/1471-2431-10-15)
Supplement: Additional file 2 — Data provision form. collection form for trial level data, data provision procedure. [file 1471-2431-10-15-S2.PDF]

MAPPiNO individual patient data meta-analysis: data provision form

Trial ID: __________________ **Name of person completing this form:** _________________________

Your email: _______________________________________ **Your fax number:** _____________________

General information about your trial

Was informed consent obtained for all patients in your trial? Yes ☐ No ☐

Date trial opened to accrual: ____ / ____ / ____ (d d / mm / y y) Date trial closed to accrual: ____ / ____ / ____ (d d / mm / y y)

At the time the trial closed to accrual, what was the total number of participants randomised? ______________

Have data for all participants randomised into the trial been published / reported? Yes ☐ No ☐

Please list **the experimental and control interventions used in each arm of your trial** (include inhaled nitric oxide concentration, duration, weaning protocol; target oxygen saturation range and target blood gas range):

Experimental arm: ____

____

Control arm: ____

____

Please describe your criteria (if any) for failure of the assigned treatment in your trial:

____

____

Please describe your criteria (if any) for permitted crossover from the assigned treatment in your trial:

____

____

Please describe the surfactant replacement therapy regime used in your trial:

____

____

Please describe your criteria (if any) for postnatal treatment with systemic corticosteroids in your trial:

____

Definitions used in your trial

Please supply information regarding how you defined the following outcomes in your trial.

Chronic lung disease: ____

Pulmonary air leak: ____

Please turn over for further information: →

Please return to: **Angela Carberry, MAPPiNO Collaboration, NHMRC Clinical Trial Centre, Locked Bag 77, Camperdown, NSW, 2050, Australia**

Email: **mappino@ctc.usyd.edu.au** **Fax: +61 (2)9565 1863** **Phone: +61 (2)9562 5000**
Ftp site: ftp://mappino@ftp.ctc.usyd.edu.au **Direct: +61 (2)9562 5028**

MAPPiNO individual patient data meta-analysis: data provision form

Definitions used in your trial (continued)

Cerebral palsy: ____

Blindness: ____

Deafness: ____

Major developmental delay: ____

____

Time point when development was assessed: ____

Respiratory outcomes at follow-up: ____

Information about the design of your trial

What method was used to generate the random allocations in the trial?

Simple (such as coin toss) ☐ Random number tables ☐ Permuted blocks ☐ Minimisation ☐

Other ☐ If other, please state method used: ____

What, if any, stratification factors were used? ____

What proportions was the trial designed to have in each arm? (for example, 1:1) ______________________

What method was used to conceal the random allocation?

Opaque, sequentially numbered, sealed envelopes ☐ Central telephone randomisation ☐

Other ☐ If 'Other', please state method used: ____

Was masking/blinding used? Yes ☐ No ☐ If 'Yes', who was masked / blinded? *(tick all that apply)*

☐ Infant's family ☐ Clinicians ☐ Outcome assessor(s) ☐ Data analysts / statisticians

Did an independent external Data Safety Monitoring Committee monitor the trial? Yes ☐ No ☐

Data transfer information

Please provide the data listed on the attached sheet, on all participants randomised, if you collected these variables in your trial. This includes any participants randomised whose data did not appear in published reports, regardless of whether or not they were included in the analyses.

Data can be supplied in almost any format (Excel, Access, Dbase, FoxPro, ASCII etc), but please indicate below which format has been used. It would be helpful if you used the coding suggested in the attached document, however, you may code the data in the way that is most convenient to you. **Please supply us with full details of the data coding system used.** If you are able to supply the full trial protocol and/or sample blank data collection sheets, this would also be very helpful.

What format have you supplied data in? ____

Please turn over for further information: →

Please return to: **Angela Carberry, MAPPiNO Collaboration, NHMRC Clinical Trial Centre Locked Bag 77, Camperdown, NSW, 2050, Australia**

Email: mappino@ctc.usyd.edu.au **Fax: +61 (2)9565 1863** **Phone: +61 (2)9562 5000**
Ftp site: ftp://mappino@ftp.ctc.usyd.edu.au **Direct: +61 (2)9562 5028**

MAPPiNO individual patient data meta-analysis: data provision form

Data transfer information (continued)

Have you used the suggested coding for the data you are supplying? Yes ☐ No ☐

If no, have you sent us details of the coding system you have used? Yes ☐ No ☐

Have you sent us a full trial protocol? Yes ☐ No ☐

Have you sent us a copy of blank data collection sheets? Yes ☐ No ☐

You can lodge your data set on a secure ftp website at the Data Coordination Centre (see below for instructions). However, please send us your data by any of the following methods if this is more convenient: completing new data forms (provided on request); sending either the original data forms, a computer printout of the data or the data files on floppy disk (formatted for PC) or CD; or by emailing the data files to us. If you email the data file(s), please encrypt the data if possible and send the encryption key to us in a separate email.

What method of data transfer have you used?

Lodged data file(s) on ftp website ☐ Emailed data file(s) ☐ Sent data file(s) on disk via post ☐

Sent computer printout of data ☐ Sent original data forms ☐ Other: ______________________

What date did you send / lodge your data? ____ / ____ / ____
d d mm y y

What date did you email us to notify re data lodgement? ____ / ____ / ____
d d mm y y

To lodge your data on the secure MAPPiNO ftp website:

Start Internet Explorer (or your web browser). Go to the website: ftp://ftp.ctc.usyd.edu.au

When prompted, enter the user name **mappino** and password **XmappinoX**. You will then see other files that have already been lodged (but you won't be able to access them). Reduce the size of the browser window that is displaying the MAPPiNO ftp website and open Windows Explorer. Size both windows so that you can see the contents of each.

Please name your file so it can be recognised as belonging to your trial, for example: Smith 2004.xls. To deposit your file, go to the appropriate folder in Windows Explorer, select it, and holding the left mouse key down (or right mouse for left hand users), drag and drop the file onto the ftp site. This will copy your file to the secure website. Close down the web browser and Windows Explorer. **Please notify us by email when you lodge your data on the ftp website.**

Guarantee of confidentiality of individual trial results

Any data supplied will remain the property of the trialist(s) who supplied it. These data will remain confidential and will not be used, circulated or distributed in any way that allows access to individual trial data.

I wish my data to remain confidential Yes ☐ No ☐

Signed ____ Date ______________________________

Please return to: **Angela Carberry, MAPPiNO Collaboration, NHMRC Clinical Trial Centre Locked Bag 77, Camperdown, NSW, 2050, Australia**

Email: mappino@ctc.usyd.edu.au **Fax: +61 (2)9565 1863** **Phone: +61 (2)9562 5000**
Ftp site: ftp://mappino@ftp.ctc.usyd.edu.au **Direct: +61 (2)9562 5028**
